# Natural Sesquiterpene Lactones of the 4,15-*iso*-Atriplicolide Type are Inhibitors of Trypanothione Reductase

**DOI:** 10.3390/molecules24203737

**Published:** 2019-10-16

**Authors:** Mairin Lenz, R. Luise Krauth-Siegel, Thomas J. Schmidt

**Affiliations:** 1 Institute of Pharmaceutical Biology and Phytochemistry (IPBP), University of Münster, PharmaCampus Corrensstraße 48, D-48149 Münster, Germany; m_lenz01@uni-muenster.de; 2 Biochemie-Zentrum der Universität Heidelberg (BZH), Im Neuenheimer Feld 328, D-69120 Heidelberg, Germany; luise.krauth-siegel@bzh.uni-heidelberg.de

**Keywords:** sesquiterpene lactone, 4,15-iso-atriplicolide ester, *Trypanosoma brucei*, *Trypanosoma cruzi*, trypanothione reductase, irreversible inhibitor, antitrypanosomal activity

## Abstract

In the course of our investigations on the antitrypanosomal potential of sesquiterpene lactones (STL), we have recently reported on the exceptionally strong activity of 4,15-*iso*-Atriplicolide tiglate, which demonstrated an IC_50_ value of 15 nM against *Trypanosoma brucei rhodesiense*, the etiologic agent responsible for East African human trypanosomiasis (HAT). Since STLs are known to often interact with their biological targets (e.g., in anti-inflammatory and anti-tumor activity) by means of the covalent modification of biological nucleophiles—most prominently free cysteine thiol groups in proteins—it was a straightforward assumption that such compounds might interfere with the trypanothione-associated detoxification system of trypanosomes. This system heavily relies on thiol groups in the form of the dithiol trypanothione (T(SH)_2_) and in the active centers of enzymes involved in trypanothione metabolism and homeostasis. Indeed, we found in the present study that 4,15-*iso*-atriplicolide tiglate, as well as its structural homologues, the corresponding methacrylate and isobutyrate, are inhibitors of trypanothione reductase (TR), the enzyme serving the parasites to keep T(SH)_2_ in the dithiol state. The TR inhibitory activity was demonstrated to be time-dependent and irreversible. Quite interestingly, of the several further STLs with different core structures that were also tested, none inhibited TR at a significant level. Thus, the TR inhibitory effect by the 4,15-*iso*-atriplicolide esters appears to be specific for this particular type of furanoheliangolide-type STL. Some structure–activity relationships can already be deduced on the basis of the data reported here, which may serve as the starting point for searching further, possibly more potent, TR inhibitors.

## 1. Introduction

Sesquiterpene lactones (STLs) have, in many cases, been found to possess antiprotozoal activity. African trypanosomes (*Trypanosoma brucei* spp.), responsible for human African trypanosomiasis (HAT) appear to be particularly sensitive to some STLs [1,2,3]. Thus, the first compound of this class ever reported as a trypanocidal agent, helenalin, possesses an in vitro IC_50_ value of only 50 nM against *T. brucei rhodesiense* (*Tbr*, causing East African HAT) [4] and still ranges among the most active STLs against this parasites. More recently, we discovered several STLs of the furanoheliangolide type as potent trypanocides, namely, budlein A, goyazensolide and, most importantly, 4,15-iso-Atriplicolide tiglate [2] with IC_50_ values in a similar range or even lower than that of helenalin. The latter compound displayed an IC_50_ value of only 15 nM, and is currently the STL with the strongest anti-*Tbr* activity discovered so far. Its methacryalate and isobutyrate analogues were more recently found to be only a little less active against this parasite [5]. Sesquiterpene lactones exert many of their biological activities by means of Michael-type additions of their reactive structure elements (mostly enone systems such as α,β-unsaturated lactone and ketone structures) to nucleophilic groups of their biological targets. Free cysteine thiol groups are most prominently affected by such modifications. Therefore, it was straightforward, already in our very first report on antitrypanosomal activity of STLs [4], to hypothesize that the activity of such compounds against the parasites under study might be due to interference with the trypanosomes’ peculiar intracellular thiol, trypanothione, and the associated enzymatic pathways needed for redox homeostasis and the detoxification of reactive species.

Unlike other eukaryotes, trypanosomatids use the bis-glutathionyl spermidine trypanothione T(SH)_2_ to maintain redox homeostasis and also for the detoxification of electrophilic xenobiotics. To maintain T(SH)_2_ in the reduced state, they utilize the enzyme trypanothione reductase (TR), which is parasite-specific and thus a potential drug target [6,7].

After the initial finding that helenalin is a potent antitrypanosomal agent, the compound was tested for a possible inhibitory activity on TR but was found to be inactive [8]. The recent discovery of furanoheliangolides with strong antitrypanosomal activity [2,5] renewed our interest in components of the trypanothione system as possible targets of such compounds. Therefore, such compounds as well as some representatives of other STL types with proven activity against *Tbr* were investigated for potential activity against TR.

## 2. Results and Discussion

### 2.1. Activity of Antitrypanosomal STLs against Trypanothione Reductase from Trypanosoma brucei (TbTR) and T. cruzi (TcTR)

**Figure 1:**
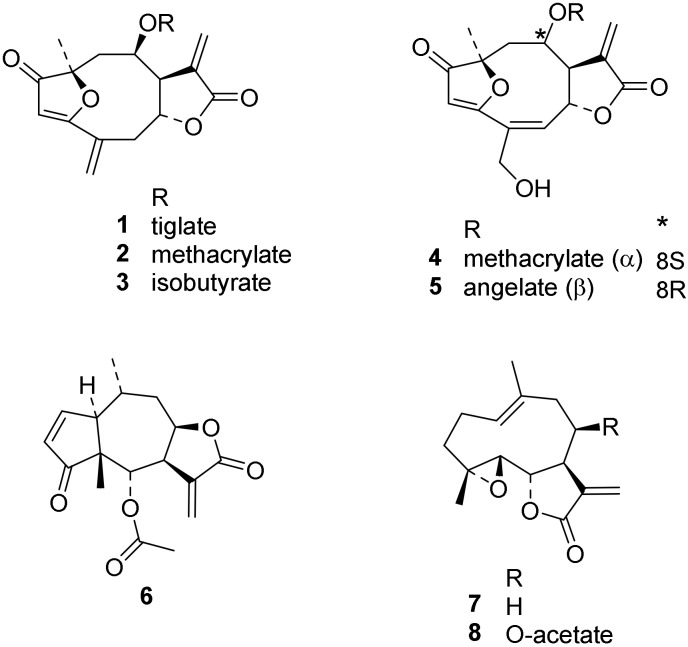
Chemical structures of the sesquiterpene lactones (STLs) tested in this study.

The antitrypanosomal activity of the STLs under study (structures shown in Figure 1) has been published previously. With IC_50_ values <0.1 µM, the iso-atriplicolide esters **1** and **2** [2,5], goyazensolide **4**, and budlein A **5** [2], as well as the helenanolide **6** [1] are among the most active STLs against *T. brucei rhodesisense* found so far. The other compounds, **3** [5], **7** [1], and **8** [2] also showed considerable activity against this parasite. All compounds were less active against *T. cruzi*, but still showed significant activity [1,2,5]. In order to obtain insights into their possible mechanism of action, their activity against trypanosomal trypanothione reductases (TR) was tested.

All the selected STLs mentioned above were submitted to inhibitory studies on recombinant TR from both *T. brucei* (*Tb*TR) as well as *T. cruzi* (*Tc*TR). The latter is structurally very similar to the enzyme of *T. brucei* (*Tb*) with 83% identical residues overall and 100% identity of the amino acids of the active site [9], so that it was included in order to test for possible differences in susceptibility. All compounds were tested against both enzymes at a concentration of 100 µM. The readout of the TR assay [10] is NADPH consumption measured by the decrease of absorption at 340 nm. The results are reported in Table 1.

**Figure 2:**
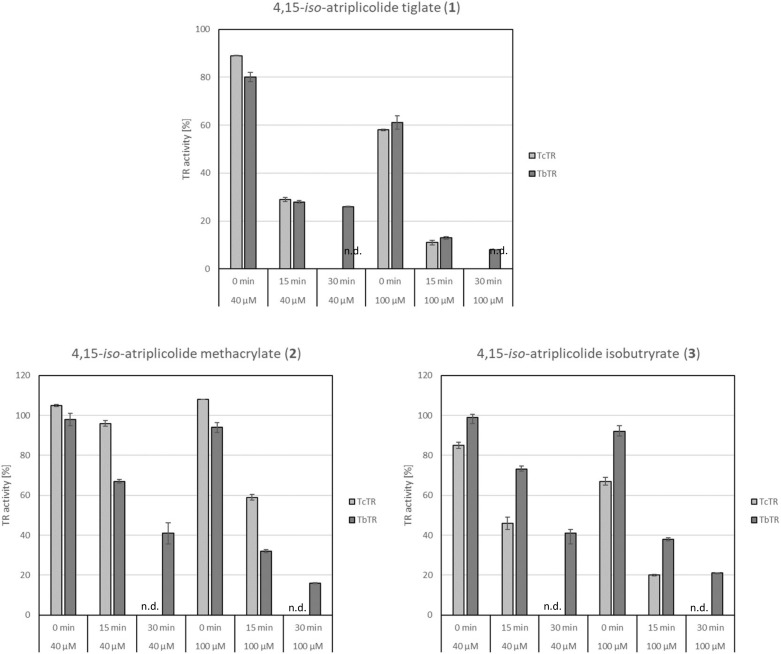
Inhibition of TR by STLs **1**–**3** in the presence of NADPH. Diagrams show the relative activity (% residual activity in relation to untreated controls) of *Tc*TR and *Tb*TR after the addition of each STL at the specified concentration and preincubation for the specified time. n.d.: Inhibition of *Tc*TR after 30 min preincubation was not determined.

Quite strikingly, only the iso-atriplicolide esters **1**–**3**, but none of the other tested STLs, showed activity against either TR under the chosen conditions. Since it was expected that the STL inhibitors would act by a covalent (i.e., irreversible) modification of the enzyme, more detailed experiments were conducted with the structural analogues **1**–**3**, comparing their effects at 40 and 100 µM concentration without and with preincubation in the presence of NADPH for 15 min as well as 30 min in case of *Tb*TR. The results are reported in Table 1 and plotted in Figure 2. Clearly, the degree of inhibition strongly increased in all cases with the preincubation time. Compound **1** turned out to be the most potent inhibitor. At 100 µM and 15 min preincubation, it inhibited *Tb*TR by 87% and *Tc*TR by 89% of the enzyme activity. All three compounds displayed a time- and concentration-dependent inhibitory activity on both *Tb*TR and *Tc*TR; however, these showed some differences in sensitivity. While the tiglate **1** is the most active inhibitor in both cases, it is noteworthy that the methacrylate **2** is a more potent inhibitor of *Tb*TR than the isobutyrate **3**, whereas it was vice versa in the case of *Tc*TR. The two enzymes share a highly conserved active site in which all residues making direct contact with the substrate are identical. Nevertheless, some differences exist that should account for the different susceptibility to inhibitors, which has previously been described for other inhibitors [11].

On the backdrop that all tested compounds are rather potent trypanocides and share the possibility of forming covalent bonds with their potential target proteins, it appears rather noteworthy that none of the other compounds (**4**–**8**) showed any measurable effect on either TR at the chosen concentration. Even the strong trypanocide helenalin acetate (**6**) was found to be essentially inactive against both TRs. At 200 µM and after 30 min preincubation, **6** caused only a minute inhibition of *Tb*TR of about 10%. Similarly, neither of the other furanoheliangolides (**4**, **5**) or germacranolides (**7**, **8**) at 100 µM showed any significant effects on *Tc*TR and *Tb*TR (neither without nor with preincubation for 30 min), thus confirming that the inhibitory activity on TR is a unique feature of the iso-atriplicolides within the tested set of STLs.

The time-dependence of the inhibitory effect of **1**–**3** gives a clear hint that the enzyme is indeed inhibited by an irreversible mechanism.

### 2.2. Mode of Inhibition of TbTR by 4,15-iso-Atriplicolide Esters

**Figure 3:**
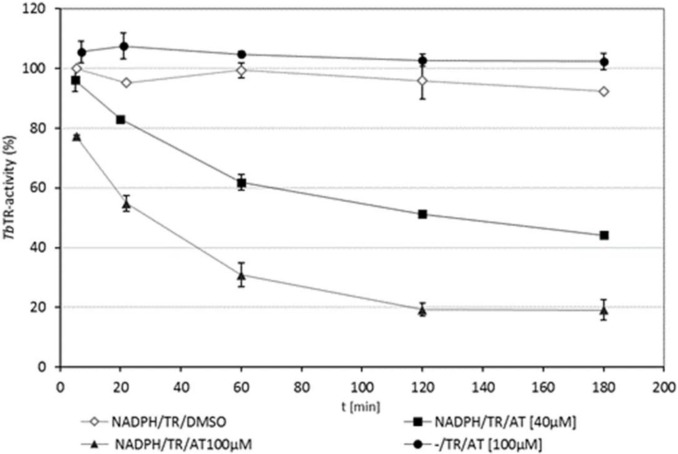
Time-dependent inhibition of *Tb*TR by STL **1** (AT). The solvent (blank control, open diamond) or the STL (40 [filled square] and 100 µM [filled triangle]) was incubated with TR in the presence of NADPH; the STL was also incubated with TR in the absence of NADPH (filled circle). Samples were taken at the different time points, and the remaining activity was measured in a standard TR assay containing 100 µM NADPH and 100 µM TS_2_ (data represent the averages of two independent determinations ± deviations from the mean).

The time-dependence of the 4,15-iso-atriplicolides’ inhibitory effect on TR already indicated that these STLs act by the covalent modification of the enzyme. In order to prove that this is indeed the case, experiments were conducted with **1**, the strongest inhibitor. *Tb*TR was preincubated with the compound in the presence/absence of NAPDH. After certain time intervals up to 3 h, 5-µL aliquots were taken, and the remaining activity was determined in a standard assay. In case of a reversible inhibitor, the inhibitory effect would be strongly diminished or even lost under these conditions, due to the 200-fold dilution of the inhibitor in the assay. Moreover, there should be no increase of the inhibitory effect over time. The results are shown in Figure 3.

It becomes evident that the inhibitory effect, also after 200-fold dilution of the aliquot used for each assay, increases over time and follows an exponential trend that is well in line with (pseudo) first-order kinetics, as expected for a chemical reaction between the enzyme and inhibitor under the chosen conditions with a high excess of inhibitors (40 and 100 µM) over enzymes (1.23 µM). The two curves also clearly show the concentration-dependence of the inhibitory activity. Analogous experiments were conducted with the congeneric esters **2** and **3**, which behaved in essentially the same manner (see Supplementary Figure S1).

### 2.3. TbTR is Only Inhibited by 4,15-iso-Atriplicolide Esters in Its Reduced Dithiol State

The active site of TR contains a redox active dithiol/disulfide couple (Cys 52 and 57). Upon the binding of NADPH to TR, the disulfide bridge is reduced, and Cys52 directly reacts with the oxidized trypanothione [7]. As Cys52 has been shown to be the target of a variety of irreversible inhibitors [12,13,14], it was straightforward to expect that this thiol group is the actual target of the Michael-type addition with the iso-atriplicolides. As can be seen in Figure 3 (filled circles), in the absence of NADPH, the enzyme indeed retains its full activity. This strongly suggests that Cys 52 is the target site for covalent modification by **1**; the same was found in analogous experiments with compounds **2** and **3** (Supplementary Figure S1).

### 2.4. Kinetics of Inhibition of TbTR by 4,15-iso-Atriplicolide Esters

**Figure 4:**
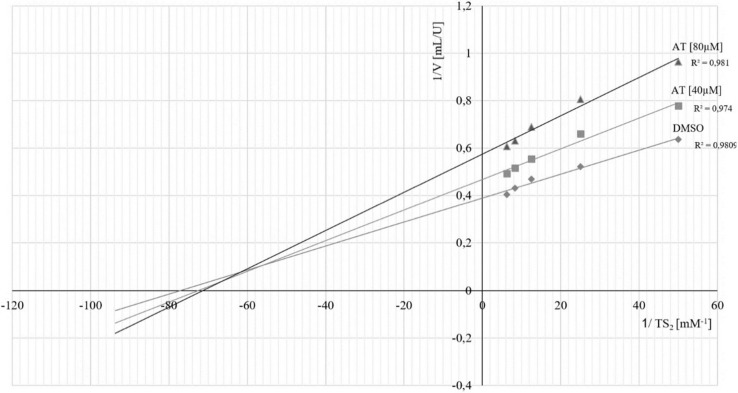
Lineweaver–Burk diagram for inhibition kinetics of compound **1** (AT) on *Tb*TR.

To further characterize the mode of inhibition, the activity of TR was measured as a function of substrate concentration, in the absence and presence of 40 and 80 µM of **1**. The Lineweaver–Burk plot (i.e., 1/v_0_ versus 1/[S], see Figure 4) showed an intersection of the three lines above the ordinate. The apparent Ki and Ki’ values were determined as 128 ± 8 and 183 ± 15 µM.

This apparent mixed inhibition type indicates that the inhibitor binds more easily to the free enzyme than to the enzyme–substrate complex [15], which is likely in the present case, where the STL must bind to the free enzyme in order to deactivate it. In other words, the small increase of K_M_ caused by an increasing concentration of **1** can be expected as a consequence of a (quite weak) competitive contribution to the overall inhibition. Such a competitive component should indeed exist, since the STL must first bind to the active site of the enzyme to form an initial, non-covalent complex, before the actual reaction occurs. In this process, it competes with the substrate, TS_2_, and it can also be displaced by increasing the substrate concentration from this initial complex, which straightforwardly explains the observed kinetics.

## 3. Materials and Methods

### 3.1. Compounds under Study

All compounds under study were previously described, and their antitrypanosomal activity was reported: 1, 4, 5, 8 [2], 2, 3 [5], 6, 7 [1].

### 3.2. Enzmes

*Tc*TR and *Tb*TR were purified from recombinant *E. coli*, as described previously [11].

### 3.3. Enzyme Inhibition Assays

Standard TR assay: The assay was carried out as described previously [10,11]. In short, to 1 mL of assay solution at pH 7.5 containing 40 mM HEPES, 1 mM EDTA, 100 µM NADPH, and about 10 mU *Tb*TR or *Tc*TR (5 µL of a stock solution containing 2 U/mL), the adequate volume of a 4-mM stock solution of the test compound in DMSO was added to yield the final specified concentration of the test compound and a concentration of 5% DMSO. 5% DMSO served as blank control. The components were mixed in a photometric cuvette thermostated at 25 °C. The cuvette was either immediately (no preincubation) or after different times (15 or 30 min preincubation) inserted into the spectrophotometer at 25 °C, and a baseline was recorded for 90 s at λ = 340 nm. Then, the reaction was started by adding trypanothione disulfide (TS_2_) [16] in a final concentration of 100 µM. Thus, both the substrate and cosubstrate were present at concentrations well above their respective K_M_ values (≈10 and ≈1 µM [10,17]), ensuring that the assay was conducted under full enzyme saturation. The absorbance at 340 nm was monitored for at least 120 s. The decrease in absorbance over time (ΔA/min) was determined from the linear first 60 s of the time course. As a measure of enzyme activity, volume activity (a_v_ [U/mL]) was determined as follows, using the millimolar absorbance coefficient of NADPH; ε(NADPH) = 6.2 mM^−1^ × cm^−1^.

$$a_{v}\;\left[\frac{U}{mL}\right]=\frac{\Delta A/min}{\varepsilon \left(NADPH\right)*d\;\left[cm\right]}\;\times \frac{total\;assay\;volume\;\left[mL\right]}{volume\;enzyme\;solution\;\left[mL\right]}$$

Each data point (enzyme activity in the presence of test compound at a specified concentration) was measured at least twice. The percentage of inhibition as reported in Table 1 was then calculated as 100 × (1 − (*a*/*b*)), where *a* is a_v_ in the presence of the test compound, and *b* in the absence of the test compound.

The enzyme kinetics to determine the type of inhibition were performed under the same conditions at three different inhibitor concentrations: 0, 40, and 80 µM (all with a DMSO concentration of 5%). Assays were performed with five different concentrations of TS_2_ over the range of 20–160 µM (20, 40, 60, 80, 120, and 160 µM). The resulting data were for volume activity were plotted in reciprocal form (mL/U) versus the reciprocal substrate concentration ([TS_2_]^−1^ (µM^−1^)) to yield the plot presented in Figure 4. Inhibition constants (Ki and Ki’ values) were determined from the slopes, and ordinate intercepts of the two data series were obtained in the presence of **1** [15].

A dilution assay to test for the irreversible inhibition of *Tb*TR: In this case, 300 mU TR were preincubated in a volume of 100 µL with 40 or 100 µM of the inhibitor in the presence or absence of 100 µM NADPH at 25 °C for up to 3 h. 5% DMSO was used instead of the STL as blank control. At various time points (5, 20, 60, 120, and 180 min), aliquots of 5 µL (15 mU TR) were taken from the mixture and added into a standard TR assay. The assay was started by adding 100 µM TS_2_, the absorbance at 340 nm was monitored as above, and the enzyme activity was determined from ΔA/min as above. The resulting remaining activity was plotted versus time (min) in Figure 3.

## 4. Conclusions

The inhibitory activity of the 4,15-*iso*-atriplicolide esters on TR appears to be unique among the tested STLs, and hence cannot be a mere consequence of the presence of reactive Michael acceptor structures. The inactivity of two other furanoheliangolides, goyazensolide and budlein A, which are structurally very similar to the *iso*-atriplicolides, leads to the conclusion that the *iso*-atriplicolide scaffold with an exocyclic double bond between C-4 and C-15 enables these compounds to bind to the active center of TR in a specific manner. It can be speculated that this double bond, as part of an α,β,γ,δ-unsaturated keto function, is actually the reactive partial structure that leads to the covalent deactivation of the enzyme. It is interesting to note that the small differences in the ester moiety confer different potency to the compounds against the TRs of *T. brucei* and *T. cruzi*. Thus, this part of the molecules has a modulatory influence on activity, and it will be interesting to investigate further ester derivatives with the same basic skeleton to study the structure–activity relationships in more detail. On the background that several STLs of this study that previously were shown to have very strong antitrypanosomal activity were inactive against TR, it can safely be stated that TR inhibition can be ruled out as a general mechanism of action of STLs against *T. brucei*. This is in line with the previous results of our group as well as those of others [8,18]. The level of inhibitory potency of the *iso*-atriplicolide esters against TR is much too low to account—on its own—for these STLs’ high-level antirypanosomal activity, for which other mechanisms must be held responsible. However, the discovery of the 4,15-*iso*-atriplicolide scaffold as a TR inhibitor opens the possibility to search for further, perhaps more potent, TR inhibitors with similar molecular characteristics.

## Figures and Tables

**Table 1:** In vitro activity of selected STLs (structures see Figure 1) against recombinant trypanothione reductase from both *T. brucei* (*Tc*TR) and *T. cruzi* (*Tb*TR). In vitro activity data against *T. brucei rhodesiense (Tbr)*, *T. cruzi (Tc)*, and L6 rat skeletal myoblasts are from our previous reports [1,2,5] and repeated here for easier comparison. Enzyme inhibition data represent mean % inhibition at 100 µM ± SD (*n* ≥ 2).

Compound	*Tbr*	*Tc*	*L6*	*Tc*TR		*Tb*TR		
	IC_50_ (µM)			% Inhibition at *c* = 100 µM Preincubation Time (min)				
				0	15	0	15	30
**1**	0.015	3.7	1.2	42 ± 0	89 ± 1	39 ± 3	87 ± 1	92 ± 0
**2**	0.077	1.6	0.52	0 ± 0	41 ± 1	6.0 ± 2.4	68 ± 1	84 ± 0
**3**	0.26	3.1	0.88	33 ± 2	80 ± 0	8.0 ± 2.8	62 ± 1	79 ± 0
**4**	0.073	1.1	0.49	n.i.	n.t.	n.t.	n.t.	n.i.
**5**	0.072	1.8	0.38	5 ± 8	n.t.	n.t.	n.t.	3 ± 3
**6**	0.063	0.54	0.81	n.i.	n.t.	n.t.	n.t.	n.i *
**7**	0.39	11	7.2	n.i.	n.t.	n.t.	n.t.	2 ± 8
**8**	0.23	11	4.7	n.i.	n.t.	n.t.	n.t.	n.i.

* no inhibition at 100 µM; 10 ± 2% inhibition observed at 200 µM of **6**; n.i.: No inhibition. n.t.: Not tested.

## Supplementary Materials

The following are available online, Figure S1: Time-dependent inhibition of *Tb*TR by STLs **2** and **3**. Analogous plot to Figure S3.
