# Supplementary material for: Natural Sesquiterpene Lactones of the 4,15-iso-Atriplicolide Type are Inhibitors of Trypanothione Reductase
Source: Molecules. 2019 Oct 16;24(20):3737. doi: 10.3390/molecules24203737 (PMC6832266; doi:10.3390/molecules24203737)
Supplement: Supplementary file 1 [file molecules-24-03737-s001.pdf]

molecules

Supplementary Data

Natural Sesquiterpene Lactones of the 4,15-*iso*-Atriplicolide Type are Inhibitors of Trypanothione Reductase

Mairin Lenz,[1] R. Luise Krauth-Siegel,[2] Thomas J. Schmidt[1,*]

[1] Institute of Pharmaceutical Biology and Phytochemistry (IPBP), University of Münster, PharmaCampus Corrensstraße 48, D-48149, Münster, Germany; thomschm@uni-muenster.de (T.J.S.); m_lenz01@uni-muenster.de (M.L.)

[2] Biochemie-Zentrum der Universität Heidelberg (BZH), Im Neuenheimer Feld 328, D-69120 Heidelberg Germany; E-mail: luise.krauth-siegel@bzh.uni-heidelberg.de (R.L.K-S).

* Correspondence: thomschm@uni-muenster.de; Tel.: +49-251-83-33378

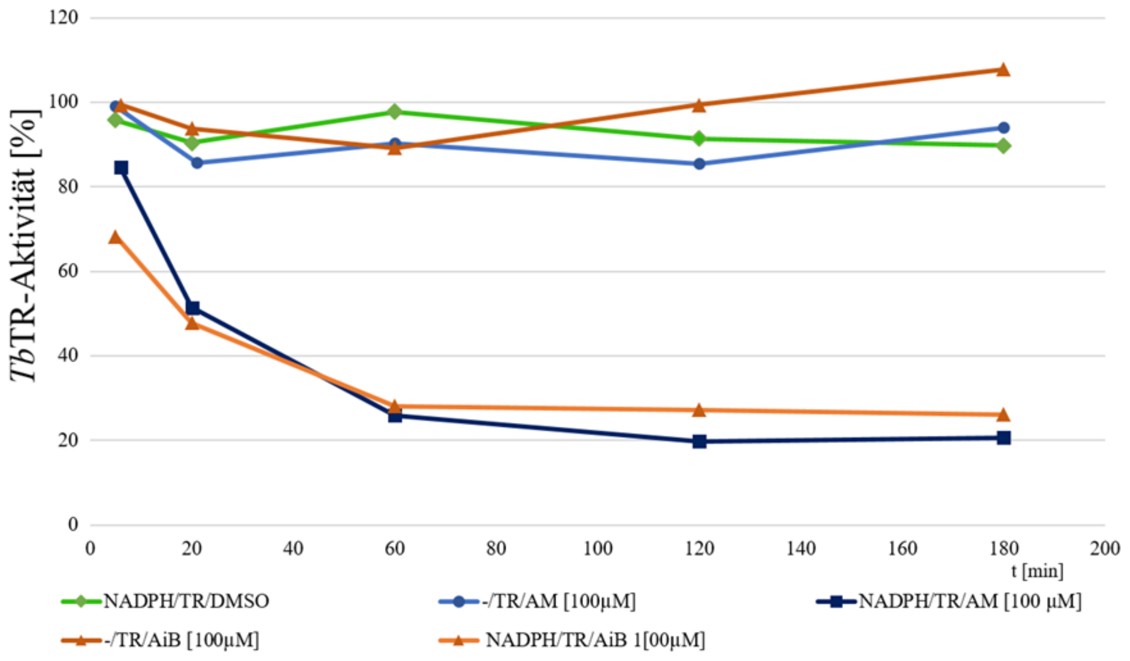

Figure S1. Time-dependent inhibition of *Tb*TR by STLs **2** (AM) and **3** (AiB). The solvent (blank control, green diamond) or STL **2** (100 µM [dark blue square]) or STL 3 (100 µM [orange triangle]) was incubated with TR in the presence of NADPH; both STL **2** and **3** were also incubated at 100 µM with TR in the absence of NADPH (light blue circle and red triangle, respectively), which was then added immediately before the enzyme activity test. Samples were taken at the different time points, diluted 200-fold and the activity of TR measured. (Data represent averages of three independent determinations; error bars omitted for clarity; all relative standard deviations were ≤ 11%).
